# Validation study of obstetric hospitalization data held on the Brazilian National Health System Hospital Information System for maternal morbidity surveillance: Brazil, 2021-2022

**DOI:** 10.1590/S2237-96222024v33e20231252.en

**Published:** 2024-07-29

**Authors:** Rosa Maria Soares Madeira Domingues, Lana dos Santos Meijinhos, Luis Carlos Torres Guillen, Marcos Augusto Bastos Dias, Valéria Saraceni, Rejane Sobrinho Pinheiro, Natália Santana Paiva, Cláudia Medina Coeli

**Affiliations:** 1 Fundação Oswaldo Cruz, Laboratório de Pesquisa Clínica em DST/Aids, Rio de Janeiro, RJ, Brazil; 2 Universidade Federal do Rio de Janeiro, Instituto de Estudos em Saúde Coletiva, Rio de Janeiro, RJ, Brazil; 3 Fundação Oswaldo Cruz, Instituto Nacional de Saúde da Mulher, da Criança e do Adolescente “Fernandes Figueira”, Rio de Janeiro, RJ, Brazil; 4 Secretaria Municipal de Saúde do Rio de Janeiro, Superintendência de Vigilância em Saúde, Rio de Janeiro, RJ, Brazil

**Keywords:** Validation Study, Morbidity, Pregnancy, Postpartum Period, Hospital Information Systems, Statistical Databases, Estudio de Validación, Morbilidad, Embarazo, Período Posparto, Sistemas de Información en Hospital, Bases de Datos Estadísticos

## Abstract

**Objective:**

To validate the Brazilian National Health System Hospital Information System (SIH/SUS) for maternal morbidity surveillance.

**Methods:**

This was a cross-sectional study conducted in 2021/2022, taking as its reference a national study on maternal morbidity (MMG) conducted in 50 public and 28 private hospitals; we compared SIH/SUS and MMG data for hospitalization frequency, reason and type of discharge and calculated sensitivity, specificity, positive and negative likelihood ratios for seven diagnoses and four procedures.

**Results:**

Hospitalizations identified on SIH/SUS (32,212) corresponded to 95.1% of hospitalizations assessed by MMG (33,867), with lower recording on SIH/SUS (85.5%) for private hospitals [10,036 (SIH/SUS)]; 11,742 (MMG)]; compared to MMG, SIH/SUS had a lower proportion of hospitalizations due to “complications during pregnancy” (9.7% versus 16.5%) as well as under-recording of all diagnoses and procedures assessed, except “ectopic pregnancy”.

**Conclusion:**

Better recording of diagnoses and procedures on SIH/SUS is essential for its use in maternal morbidity surveillance.

## Study contributions

**Main results:**

The SIH/SUS system had good recording coverage of obstetric hospitalizations, but a lower proportion of hospitalizations due to “complications during pregnancy”; and under-recording of the diagnoses and procedures assessed, except “ectopic pregnancy”.

**Implications for services:**

The quality of hospital admission authorization (AIH) filling in needs to be improved, including recording diagnoses and procedures to identify hospitalization as obstetric hospitalization as well as complications in pregnancy and the postpartum period.

**Perspectives:**

Improving the quality of AIH records is essential for the use of the SIH/SUS system in maternal morbidity surveillance, this being a strategy recommended for better obstetric care, being complementary to maternal mortality surveillance.

## RESUMEN

**Objetivo:**

Validar el Sistema de Información Hospitalaria del Sistema Único de Salud (SIH/SUS) para vigilancia de la morbilidad materna.

**Métodos:**

Estudio transversal, 2021/2022, utilizando como referencia datos de estudio nacional de morbilidad materna (MMG) realizado en 50 hospitales públicos y 28 privados; comparando: frecuencia, motivo y tipo de alta de internaciones en SIH/SUS y MMG y calculando sensibilidad, especificidad y razones de probabilidad positivos y negativos para siete diagnósticos y cuatro procedimientos.

**Resultados:**

Las internaciones identificadas en SIH/SUS (32.212) correspondieron al 95,1% de internaciones evaluadas en MMG (33.867), observándose menor registro en SIH/SUS (85,5%) en hospitales privados [10.036 (SIH/SUS)]; 11.742 (MMG)]; comparado con MMG, SIH/SUS tuvo menor proporción de internaciones por “complicaciones durante el embarazo” (9,7% vs 16,5%), así como subregistro de todos los diagnósticos y procedimientos evaluados, excepto “embarazo ectópico”.

**Conclusión:**

Mejor registro de diagnósticos y procedimientos en SIH/SUS es fundamental para su uso en la vigilancia de la morbilidad materna.

## INTRODUCTION

Maternal mortality is a serious public health problem in Brazil and worldwide.^1^ Although the Brazilian maternal mortality ratio is high, maternal death is an infrequent event, especially in places with a low number of births. Since 2011, the World Health Organization (WHO) has recommended the study of severe maternal morbidity and maternal near miss as complementary strategies to the study of maternal death, as they are more frequent events and share the same determining factors, thus enabling more robust analyses.^2^

The objective of the Brazilian National Health System Hospital Information System (SIH/SUS) is payment of hospitalizations with public funding. Although health surveillance is not one its purposes, the SIH/SUS has been used to investigate hospital morbidity,^3^ including maternal morbidity.^4-8^ Previous studies,^9,10^ conducted in the 1990s, evaluated SIH/SUS data reliability, including information on childbirth.^11^

However, no studies were identified that have evaluated obstetric hospitalizations nationally using the SIH/SUS, when compared to hospitalization data obtained from hospital records, despite the use of administrative data to study maternal and neonatal morbidity being reported in the international literature.^12-14^ Analyzing the information available on the SIH/SUS is, therefore, relevant for confirming its validity in the study of maternal morbidity, developing strategies to improve obstetric care and reducing maternal mortality. 

In 2021-2022, the national study entitled “Perinatal mortality, severe maternal morbidity and maternal near miss” study (“*Mortalidade perinatal, morbidade materna grave e near miss materno*” – MMG study)^15^ assessed severe maternal morbidity in Brazilian public and private hospitals using medical record data. 

This study aimed to validate SIH/SUS usefulness for monitoring maternal morbidity (MM), using the MMG study as a reference standard, by comparing obstetric hospitalization frequency, reason and discharge type, and calculating sensitivity, specificity and positive and negative likelihood ratio of diagnoses and procedures recommended by WHO^2^ for such surveillance. 

## METHODS

### Study design 

This is a cross-sectional study, conducted in 2021/2022, to validate the SIH/SUS for MM surveillance using the MMG study as a reference standard.^15^

### Background

The MMG is a hospital-based study with national coverage, carried out in an integrated manner with the study entitled *Birth in Brazil II: National survey of abortion, delivery and birth* (“*Nascer no Brasil II: pesquisa nacional sobre aborto, parto e nascimento* – NBII”). All health facilities – public and private – with more than 2,750 births/year and participating in the NBII study were included in the MMG study. A obstetric hospitalization census was conducted in those facilities for 30 consecutive days in 2021-2022. 

The SIH/SUS is a nationwide health information system, composed of two databases: the reduced database, which contains hospitalization diagnoses and the primary medical procedure performed; and the professional services database, containing a record of all professional acts carried out during hospital stay. The Hospital Admission Authorization (AIH) number identifies hospitalization of the same individual in both databases. 

According to SIH/SUS standards,^16^ it is possible that, in specific situations, a woman may have more than one AIH during an obstetric hospitalization. For example, when a woman admitted for an obstetric procedure requires a surgical intervention. In such situations,^16^ the initial AIH must be closed with the “continuing inpatient stay” billing reason and a new AIH must be issued.

### Data sources 

In the MMG study, all data were derived from medical record data collection, performed in a standardized way by trained professionals. The total number of hospitalizations, reason for hospitalization, discharge type and diagnoses and medical procedures indicating MM^15^ were obtained from the triage form, a data collection instrument filled out for all women included in the study. For women with morbidity recorded on the triage form, additional data collection was performed, using a detailed instrument. 

In the case of the SIH/SUS, the total number of hospitalizations, reason for hospitalization, discharge type and hospitalization diagnoses were obtained from the reduced database, while procedures performed were obtained from both the reduced database and the professional services database. Both databases were accessed on August 15, 2022. 

### Participants

The MMG study included 78 hospitals – 50 public and 28 private, that had concluded data collection by February 2022 –, spread over all the country’s Federative Units, except Amapá. 

**Figure 1:**
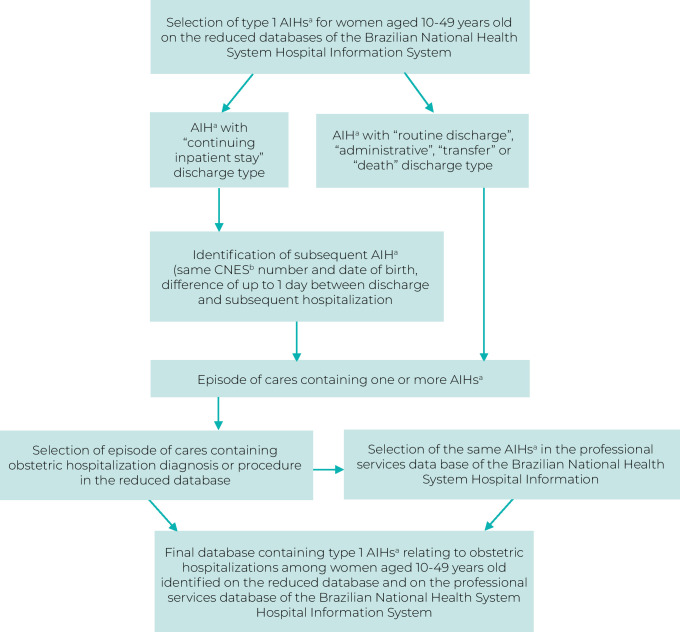
Procedures adopted for selecting obstetric hospitalizations from the Brazilian National Health System Hospital Information System, Brazil, 2021-2022

In the case of the SIH/SUS, all obstetric hospitalizations that occurred in the same period as the MMG study were included, in each of the participating hospitals, identified by their National Health Establishment Registry (CNES) number. The hospitalization selection process had four stages (Figure 1). Initially, type 1 AIHs of women aged 10 to 49 years old were selected (without identification) from the reduced database, available on the website of the SUS Information Technology Department and captured by the Microdatasus package,^17^ using R statistical programming language. 

In the second stage , we identified each woman’s hospital *episode of care*,^18^ defined as the set of all information relating to a given hospitalization, which may consist of one or more AIH. In order to identify an *episode of care* formed by multiple AIH records, an algorithm was used that allowed identification of AIHs subsequent to an AIH with the “continuing inpatient stay” billing reason. In short, AIHs with the same CNES number and for women who had the same date of birth were considered as being part of the same *episode of care*, if the interval between the discharge date of an AIH containing the “continuing inpatient stay” billing reason and the date of subsequent AIH hospitalization was less than or equal to one day. 

Subsequently, in order to identify obstetric hospitalizations, we selected AIHs with hospitalization diagnoses [according to the International Statistical Classification of Diseases and Related Health Problems – Tenth Revision (ICD-10)] and/or procedures as per the SUS *Sistema de Gerenciamento da Tabela de Procedimentos, Medicamentos e Órteses, Próteses e Materiais Especiais*)^19^ described in Box 1. In this selection process, we identified AIHs with criteria for obstetric hospitalization in *episodes of care* with single or multiple records, regardless of the position of the AIH in the episode. Finally, after identifying obstetric hospitalizations on the reduced database, AIHs with the same number were selected on the professional services database.

**Box 1:** Operational definitions used on the Brazilian National Health System Hospital Information System database for classifying obstetric hospitalization, reason for hospitalization, discharge type, specific diagnoses and procedures

**Criterion**	**ICD** ^a^		**Procedures** ^b^
**Obstetric hospitalization**	ICD group “O” recorded in one of the following fields: primary diagnosis, secondary diagnosis, secondary diagnoses 1 to 9, ICD associated, ICD notification, ICD death	OR	02.01.01.001-1, 02.11.04.001-0, 02.11.04.006-1, 03.10.01.001-2, 03.10.01.002-0, 03.10.01.003-9, 03.10.01.004-7, 03.10.01.005-5, 03.03.10.001-0, 03.03.10.002-8, 03.03.10.003-6, 03.03.10.004-4, 03.03.10.005-2, 04.09.06.001-1, 04.09.06.005-4, 04.09.06.007-0, 04.11.01.001-8, 04.11.01.002-6, 04.11.01.003-4, 04.11.01.004-2, 04.11.01.005-0, 04.11.01.007-7, 04.11.01.008-5, 04.11.02.001-3, 04.11.02.002-1, 04.11.02.003-0, 04.11.02.004-8, 04.17.01.002-8, 04.17.01.001-0, 04.17.01.003-6
**Reason for hospitalization**			
Pregnancy with abortive outcome	Primary ICD O00 to O08		
Delivery	Primary ICD 032 to 036, O60 to O69, O75, O80 to O84, P95	OR	03.10.01.001-2, 03.10.01.003-9, 03.10.01.004-7, 03.10.01.005-5, 04.11.01.002-6, 04.11.01.003-4, 04.11.01.004-2
Complications during pregnancy	Primary ICD O10 to O28, O30, O31, O40-O48 or any ICD from another group, as long as the procedure is not 03.10.01.001-2, 03.10.01.003-9, 03.10.01.004-7, 03.10.01.005-5, 04.11.01.002-6, 04.11.01.003-4, 04.11.01.004-2	OR	03.03.10.004-4
Complications related to the puerperium	Primary ICD O70, O71, O72, O73, O85 to O94		03.03.10.001-0
**Discharge type**			
Routine discharge	Billing reason = 1, 6.1, 6.2, 6.3, 6.4		
Administrative	Billing reason = 5		
Continuing inpatient stay	Billing reason = 2		
Death	Billing reason = 4, 6.5, 6.6, 6.7		
Transfer	Billing reason = 3		
Left blank	Billing reason not filled in		
**Specific diagnoses and procedures**			
Severe pre-eclampsia	O141		
Eclampsia	O150, O151, O152, O159	OR	03.03.10.002-8
HELLP^c^ syndrome	O142		
*A* *bruptio placentae*	O450, O458, O459		
Postpartum or postabortion hemorrhage	O720, O721, O722, O723, O031, O036, O041, O046, O051, O056, O061, O066, O071, O076, O081		
Rupture of uterus	O710 and O711		
Ectopic pregnancy	O000, O001, O002, O008, O009	OR	04.11.02.004-8
Hysterectomy	O822	OR	04.11.02.003-0, 04.09.06.010-0, 04.09.06.011-9, 04.09.06.012-7, 04.09.06.013-5
Laparotomy			04.07.04.016-1
Blood product transfusion	Z513	OR	03.06.02.006-8, 03.06.02.007-6, 03.06.02.008-4, 03.06.02.009-2, 03.06.02.010-6, 03.06.02.011-4, 03.06.02.012-2, 03.06.02.013-0, 03.06.02.014-9
Hospitalization in ICU^d^			UTI_MES_TO^e^ variable > 0 or procedures 08.02.01.010-5, 08.02.01.008-3, 08.02.01.009-1, 08.02.01.029-6, 08.02.01.031-8

a) ICD: International Statistical Classification of Diseases and Related Health Problems; b) Code for the procedure performed, according to the SIGTAP table (the SUS Sistema de Gerenciamento da Tabela de Procedimentos, Medicamentos e OPM); c) HELLP syndrome: Severe form of pre-eclampsia, characterized by hemolysis, elevated liver enzymes and low platelet count, in pregnant or puerperal patients; d) ICU: Intensive care unit; e) UTI_MES_TO: Number of days hospitalized in ICU.

### Variables 

The variables analyzed were:

a) Hospital type (public; private)
b) Total number of obstetric hospitalizations
c) Reason for hospitalization (delivery; abortion; complications during pregnancy; complications during the puerperium)
d) Discharge type (routine discharge; administrative; continuing inpatient stay; death; transfer)
e) Obstetric diagnoses
  – Severe pre-eclampsia
  – Eclampsia
  – HELLP syndrome (hemolysis, elevated liver enzymes, low platelet count)
  – *Abruptio placentae*
  – Postpartum/post-abortion hemorrhage
  – Rupture of uterus
  – Ectopic pregnancy
f) Hospital procedures
  – Hysterectomy
  – Laparotomy
  – Blood product transfusion
  – Hospitalization in an intensive care unit (ICU)

The seven diagnoses and four procedures listed in items “e” and “f” are part of the 26 criteria recommended by the WHO for studying severe maternal morbidity.^2^

### Detailing of the variables 

In the MMG study, hospitalizations due to “complications during pregnancy” were classified as all those due to clinical and/or obstetric complications occurring during pregnancy, without indication of termination of pregnancy at the time of hospital admission; and “complications during the puerperium” were classified as all hospitalizations resulting from clinical and/or obstetric complications diagnosed after the end of pregnancy. The “continuing inpatient stay” discharge type was considered to be a woman who remained hospitalized after the 42^nd^ day after the end of pregnancy. We used medical records to obtain data on diagnoses and procedures but did not classify them. If there were no records for these items, we considered this to be lack of diagnosis and absence of the procedure. 

In the case of the SIH/SUS, we used the criteria described in Box 1 to classify the reason for hospitalization and the discharge type. An *episode of care* in which the last AIH showed discharge due to continuing inpatient stay, and for which it was not possible to identify the subsequent AIH in that *episode of care*, was classified as “continuing inpatient stay” discharge type. In the case of *episodes of care* with more than one AIH, the “reason for hospitalization” recorded on the first AIH of the *episode of care* and the “discharge type” recorded on the last AIH of the *episode of care* were used. We used the ICD codes and procedures described in Box 1 to identify diagnoses and procedures, considering the records of all AIHs in the *episode of care*. In the case of a specific diagnosis or procedure recorded on more than one AIH of the *episode of care*, the diagnosis and/or procedure was only counted once. 

### Statistical methods

All analyses were performed comparing SIH/SUS hospitalization data (source to be validated) with hospitalization data presented in the MMG study (reference standard), for all hospitals together and for public hospitals and private hospitals separately, using version 4.3.0 of the R statistical programming language.^20^

Initially, we compared the frequency of obstetric hospitalizations, reasons for hospitalization and types of discharge identified by the MMG study and corresponding data recorded on the SIH/SUS. When comparing the total number of hospitalizations, record coverage equal to or greater than 90% was considered adequate, this being a percentage used by the Brazilian Ministry of Health as a parameter for record coverage in other information systems.^21^ The “reason for hospitalization” variable was included on the MMG study triage form after the start of fieldwork, whereby hospitals with more than 10% of hospitalizations without this variable were excluded from this analysis.

Subsequently, specific diagnoses and procedures were compared in both databases. For this analysis, only women from the MMG study who presented morbidity were included. These hospitalizations were deterministically linked to the SIH/SUS database, using the information available in both databases [CNES number; date of birth; date of admission; discharge date; race/skin color; postcode of residence]. Once the obstetric hospitalizations were linked, we compared the frequency of specific diagnoses and procedures, as well as the sensitivity, specificity, and positive (positive LR) and negative (negative LR) likelihood ratio of each diagnosis and specific procedure held on the SIH/SUS. Positive LR (sensitivity/1 - specificity) refers to presence of diagnosis; and negative LR (1 - sensitivity/specificity), refers to absence of diagnosis. Values ​​greater than 1 increase the probability of diagnosis and values ​​between 0 and 1 reduce this probability. The following criteria were used for the purposes of interpretation:

a) Positive LR: > 10 = strong; 5-9.9 = moderate; 2-4.9 = weak; 1-2 = very weak.
a) Negative LR: < 0.1 = strong; 0.11-0.20 = moderate; 0.21-0.50 = weak; 0.51-1 = very weak.^22^

All the codes used are available at: http://github.com/coelicm/SIH-SUS-validation

### Ethical considerations

The MMG study project was approved by the Research Ethics Committee of the Sergio Arouca National School of Public Health/Oswaldo Cruz Foundation, as per Opinion No. 4.230.028, issued on August 21, 2020, later amended as approved by Opinion No. 4.473.968, issued on December 18, 2020. As this was a retrospective study with data collection from medical records, waiver of signing of the Free and Informed Consent Form was requested, with access to records being authorized by the hospitals. All precautions were taken to guarantee information secrecy and confidentiality. Publicly accessible databases were used for analyzing SIH/SUS data, whereby there was no identification of the health service users in question. 

## RESULTS

**Figure 2:**
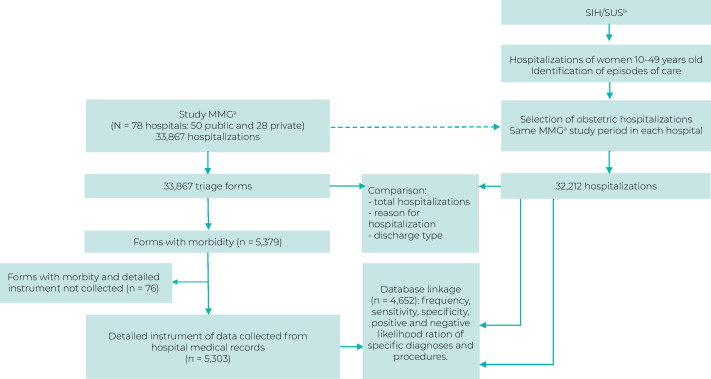
Flowchart of hospitalizations according to the Severe Maternal Morbidity study and the Brazilian National Health System Hospital Information System, Brazil, 2021-2022

The MMG study recorded 33,867 obstetric hospitalizations, of which 5,379 had a record of some MM, with detailed data being collected from 5,303 medical records. On the SIH/SUS system, 32,212 obstetric hospitalizations were identified in the same hospitals and same study period, 4,652 of which were related to hospitalizations identified by the MMG study that had diagnosis of morbidity (Figure 2).

The number of obstetric hospitalizations captured on the SIH/SUS corresponded to 95.1% of the number of hospitalizations assessed by the MMG study, with this proportion being 100.2% for public hospitals (22,176/22,125) and 85.5% for private hospitals (10,036/11,742). However, recording frequency varied between hospitals: when SIH/SUS data were compared with the MMG study data, the proportion of recording was below 90% for 8% of public hospitals and 50% of private hospitals, while the proportion of hospitalization recording was above 110% for 14% of public hospitals and 10.7% of private hospitals. 

There was a higher proportion of hospitalizations due to “complications during pregnancy” in the MMG study, both in public hospitals (16.1% versus 9.9%) and in private hospitals (17.4% versus 9.1%), and a higher proportion of admissions for delivery on the SIH/SUS (78.7% versus 72.2% in public hospitals; 81.2% versus 73.0% in private hospitals). The reasons for discharge in the MMG study and on the SIH/SUS were similar (Table 1), highlighting that “continuing inpatient stay” had different interpretations in the two databases. 

**Table 1:** Reason for obstetric hospitalization and hospital discharge type according to the Severe Maternal Morbidity study and the Brazilian National Health System Hospital Information System, Brazil, 2021-2022

**Reason for hospitalization**	**Public units**		**Private units**		**Total**	
	**MMG** ^a^ **(n = 18,688)** ^c^ **(%)**	**SIH/SUS** ^b^ **(n = 18,911)** **(%)**	**MMG** ^a^ **(n = 8,615)** ^c^ **(%)**	**SIH/SUS** ^b^ **(n = 7,177)** **(%)**	**MMG** ^a^ **(n = 27,303)** ^c^ **(%)**	**SIH/SUS** ^b^ **(n = 26,088)** **(%)**
Delivery	72.2	78.7	73.0	81.2	72.5	79.4
Abortion	9.2	8.7	7.7	7.3	8.8	8.3
Complications during pregnancy	16.1	9.9	17.4	9.1	16.5	9.7
Complications during puerperium	2.3	2.5	1.8	1.7	2.1	2.3
Other^d^	–	0.2	–	0.7	–	0.4
Left blank^e^	0.1	–	0.1	–	0.1	–
**Discharge type**	**Public units**		**Private units**		**Total**	
	**MMG** ^a^ **(n = 22,125)** **(%)**	**SIH/SUS** ^b^ **(n = 22,176)** **(%)**	**MMG** ^a^ **(n = 11,742)** **(%)**	**SIH/SUS** ^b^ **(n = 10,036)** **(%)**	**MMG** ^a^ **(n =33,867)** **(%)**	**SIH/SUS** ^b^ **(n = 32,212)** **(%)**
**Routine discharge**	96.7	97.0	98.1	99.0	97.2	97.6
Administrative	1.4	0.6	1.0	**–**	1.3	0.5
Continuing inpatient stay^f^	–	0.9	–	0.3	–	0.7
Death	–	0.1	0.1	0.1	0.1	0.1
Transfer	1.9	1.4	0.7	0.5	1.5	1.1

a) MMG: “Perinatal mortality, severe maternal morbidity and maternal near miss” study (estudo “Mortalidade perinatal, morbidade materna grave e *near miss* materno”); b) SIH/SUS: Brazilian National Health System Hospital Information System; c) Hospital units with proportion of hospitalization “not filled in” > 10% in the MMG study were excluded; d) “Other” refer to other hospitalization diagnoses not classified as delivery, abortion, complications during pregnancy or complications during puerperium; e) “Left blank” corresponds to cases with no information on the reason for hospitalization; f) In the MMG study, the “continuing inpatient stay” discharge type refers to women who remained hospitalized after the 42^nd^ day postpartum or post-abortion, while on the SIH/SUS, the “continuing inpatient stay” discharge type refers to the “continuing inpatient stay” billing reason, used when a women meets a criterion that requires the issuing of a new Hospital Admission Authorization (Autorização de Internação Hospitalar - AIH) as part of the same hospitalization, whereby the subsequent AIH was not identified.

Analysis of specific diagnoses and procedures (Table 2) showed that the most frequent diagnoses in the MMG study were “severe pre-eclampsia” (17.7%) and “hemorrhage” (13.8%), with “hospitalization in ICU” being the most frequent procedure (7.1%). On The SIH/SUS, the most frequent diagnoses were “severe pre-eclampsia” (8.9%) and “ectopic pregnancy” (4.2%); and among the procedures, the most frequent were “hospitalization in ICU” (5.3%) and “blood product transfusion” (5.2%). We highlight the absence of HELLP syndrome cases on the SIH/SUS and the identification of procedures related to “blood product transfusion” and “hospitalization in ICU” only on the professional services database.

Two diagnoses (“eclampsia” and “*abruptio placentae*”) and three procedures (“laparotomy”, “blood product transfusion” and “hospitalization in ICU”) showed a “strong” positive LR in public and private hospitals, while “severe pre-eclampsia”, “rupture of uterus” and “ectopic pregnancy” also showed a “strong” positive LR in public hospitals. “Postpartum or post-abortion hemorrhage” showed a “moderate” positive LR in public hospitals and “very weak” positive LR in private hospitals. All diagnoses and procedures showed “very weak” negative LR in public and private hospitals, except “ectopic pregnancy” (“strong” in public and private hospitals), “blood product transfusion” (“weak” in private hospitals) and “hospitalization in ICU” (“weak” in public hospitals and “moderate” in private hospitals) (Table 2). 

**Table 2:** Frequency, sensitivity, specificity and negative and positive likelihood ratios of specific diagnoses and procedures in obstetric hospitalizations, by type of hospital, according to the Brazilian National Health System Hospital Information System, Brazil, 2021-2022

**Specific diagnoses and procedures**	**Data source**					**Sens.** ^g^ **(%)**	**Spec.** ^h^ **(%)**	**LR neg** ^i^	**LR pos** ^j^
	**MMG** ^a^ **n (%)**	**SIH/SUS** ^b^							
		**ICD** ^c^ **n (%)**	**PROC_REA** ^d^ **n (%)**	**ATO PROF** ^e^ **n (%)**	**TOTAL** ^f^ **n (%)**				
**Diagnoses**									
**Severe pre-eclampsia**									
Public hospital	639 (18.3)	317 (9.1)	–	–	317 (9.1)	39.3	97.7	0.62	17.0
Private hospital	184 (15.9)	97 (8.4)	–	–	97 (8.4)	33.7	96.4	0.69	9.4
Total	823 (17.7)	414 (8.9)			414 (8.9)	38.0	97.4	0.64	14.4
**Eclampsia**									
Public hospital	94 (2.7)	71 (2.0)	4 (0.1)	4 (0.1)	71 (2.0)	20.2	98.5	0.81	13.2
Private hospital	32 (2.8)	15 (1.3)	–	–	15 (1.3)	31.3	99.6	0.69	70.3
Total	126 (2.7)	86 (1.8)	4 (0.1)	4 (0.1)	86 (1.8)	23.0	98.7	0.78	18.3
**HELLP** ^k^ **syndrome**									
Public hospital	105 (3.0)	–	–	–	–		100.0	1.0	–
Private hospital	40 (3.5)	–	–	–	–		100.0	1.0	–
Total	145 (3.1)	–			–	–	100.0	1.0	–
*Abruptio placentae*									
Public hospital	129 (3.7)	59 (1.7)	–	–	59 (1.7)	42.6	99.9	0.57	358.9
Private hospital	47 (4.1)	16 (1.4)	–	–	16 (1.4)	25.5	99.6	0.75	70.8
Total	176 (3.8)	75 (1.6)			75 (1.6)	38.1	99.8	0.62	213.0
**Hemorrhages**									
Public hospital	466 (13.3)	53 (1.5)	–	–	53 (1.5)	5.2	99.0	0.96	5.4
Private hospital	175 (15.1)	22 (1.9)	–	–	22 (1.9)	2.9	98.3	0.99	1.7
Total	641 (13.8)	75 (1.6)			75 (1.6)	4.5	98.9	0.97	3.9
**Rupture of uterus**									
Public hospital	23 (0.7)	2 (0.1)	–	–	2 (0.1)	4.3	100.0	0.96	151.0
Private hospital	1 (0.1)	1 (0.1)	–	–	1 (0.1)	–	99.9	1.0	–
Total	24 (0.5)	3 (0.1)			3 (0.1)	4.2	100.0	0.96	96.4
**Ectopic pregnancy**									
Public hospital	169 (4.8)	157 (4.5)	121 (3.5)	121 (3.5)	159 (4.5)	93.5	100.0	0.07	3,110.5
Private hospital	41 (3.5)	33 (2.9)	30 (2.6)	31 (2.7)	37 (3.2)	90.2	100.0	0.10	–
Total	210 (4.5)	190 (4.1)	151 (3.2)	152 (3.3)	196 (4.2)	92.2	100.0	0.07	4,124.7
**Specific procedures**									
**Hysterectomy**									
Public hospital	40 (1.1)	1 (0.0)	9 (0.3)	8 (0.2)	15 (0.4)	37.5	100.0	0.62	–
Private hospital	23 (2.0)	–	4 (0.3)	6 (0.5)	8 (0.7)	34.8	100.0	0.65	–
Total	63 (1.4)	1 (0.0)	13 (0.3)	14 (0.3)	23 (0.5)	36.5	100.0	0.63	–
**Laparotomy**									
Public hospital	168 (4.8)	–	28 (0.8)	28 (0.8)	28 (0.8)	16.1	100.0	0.84	534.9
Private hospital	44 (3.8)	–	6 (0.5)	6 (0.5)	6 (0.5)	11.4	99.9	0.89	126.4
Total	212 (4.6)		34 (0.7)	34 (0.7)	34 (0.7)	15.1	100.0	0.85	335.1
**Blood product transfusion**									
Public hospital	187 (5.3)	–	–	162 (4.6)	162 (4.6)	46.0	97.7	0.55	20.0
Private hospital	73 (6.3)	–	–	80 (6.9)	80 (6.9)	61.6	96.8		19.1
Total	260 (5.6)	–	–	242 (5.2)	242 (5.2)	50.4	97.5	0.51	22.4
**Hospitalization in ICU** ^l^									
Public hospital	243 (7.0)	168 (4.8)^m^	–	158 (4.5)	168 (4.8)	65.0	99.7	0.35	211.5
Private hospital	86 (7.4)	80 (6.9)^m^	–	71 (6.1)	80 (6.9)	86.0	99.4	0.14	153.5
Total	329 (7.1)	248 (5.3)^m^	–	229 (4.9)	248 (5.3)	70.5	99.6	0.30	190.5

a) MMG: “Perinatal mortality, severe maternal morbidity and maternal near miss” study (estudo “Mortalidade perinatal, morbidade materna grave e *near miss* materno”); b) SIH/SUS: Brazilian National Health System Hospital Information System; c) ICD: International Statistical Classification of Diseases and Related Health Problems; d) PROC_REA: Procedure performed recorded on the SIH/SUS reduced database; e) ATO PROF: Procedure performed recorded on the SIH/SUS professional services database; f) TOTAL: Diagnosis considering records on ICD, PROC-REA and ATO PROF; g) Sens.: Sensitivity; h) Spec.: Specificity; i) LR neg.: Negative likelihood ratio; j) LR pos.: Positive likelihood ratio; k) HELLP syndrome (severe form of pre-eclampsia, characterized by hemolysis, elevated liver enzymes and low platelet count, in pregnant or puerperal patients); l) ICU: Intensive care unit; m) Hospitalization in ICU, identified on the reduced database by means of the “UTI_MES_TO” variable, which contains information on the number of days of ICU hospitalization. Total hospitalizations in public units: 3,496, and total hospitalizations in private units: 1,156.

## DISCUSSION

The results we found showed high capture of obstetric hospitalizations on the SIH/SUS, mainly for public hospitals, with similar proportions in terms of discharge type and reason for hospitalization, in the comparison between SIH/SUS records and MMG study records; with the exception of hospitalizations due to “complications during pregnancy”, which had a 40% lower proportion on the SIH/SUS. Eight diagnoses and procedures showed a “strong” positive LR in public hospitals and five in private hospitals, demonstrating the high probability of these diagnoses and procedures when registered on the SIH/SUS. However, all diagnoses and procedures, with the exception of “ectopic pregnancy”, showed evidence of under-reporting, limiting the use of the SIH/SUS for monitoring maternal morbidity. 

The lower number of hospitalizations found for private hospitals is a result to be expected, since not all beds in SUS outsourced private hospitals provide hospitalization paid for with public funding. Possible explanations for the variation in the proportion of records found in the hospitals assessed include (i) non-issuance of AIH, (ii) non-authorization of AIH issued or even, (iii) issuance of AIH without group “O” diagnoses or procedures used to identify obstetric hospitalizations. 

In hospitalizations with primary diagnosis and procedures for non-obstetric causes, as may have occurred in hospitalizations due to COVID-19, it is essential that group “O” codes/ICDs related to complications during pregnancy or in the postpartum period be recorded in secondary diagnoses, so that such hospitalizations can be identified as having occurred during pregnancy or in the postpartum period. 

Regarding the greater number of AIHs identified on the SIH/SUS, the explanatory hypotheses include (i) non-identification of an AIH as part of an *episode of care*, being counted as hospitalization of another woman, (ii) failure to capture hospitalization of pregnant and postpartum women through the MMG study, if women diagnosed with COVID-19 were hospitalized outside the maternity ward (for example, in respiratory isolation beds), and (iii) issuance of AIH for hospitalizations that did not take place.

The findings as to a lower proportion of hospitalizations due to “complications during pregnancy” on the SIH/SUS can be attributed to the way in which the reason for hospitalization was classified on both databases: on the SIH/SUS, women diagnosed with a pregnancy complication during hospitalization were classified as “delivery” if the procedure performed was one of those provided for natural birth assistance or cesarean section. It is important to note the very similar proportions of hospitalizations for abortion care, indicating that there is no under-reporting of hospitalizations due to abortion, which would be possible, considering the illegality and stigmatization of the topic in Brazil.

The majority of discharges were routine hospital discharges, with the proportion of deaths being low and similar between the two databases. A study evaluating maternal mortality in Brazil in 2019, estimated through data registered on the SIH/SUS, concluded that use of the SIH/SUS may be valid in studies on maternal mortality and morbidity, as an information system complementary to the Mortality Information System.^23^

Under-recording of diagnoses and procedures on the SIH/SUS database has implications for the study of maternal morbidity, especially hypertensive and hemorrhagic complications, these being the most frequent causes found both in the MMG study and in national^4,6,8^ and international studies.^24,25^ Problems with recording specific diagnoses on the SIH/SUS have already been reported in previous studies.^3^

In the case of “severe pre-eclampsia” diagnosis, it is possible that other ICD codes related to hypertensive complications may have been used, reflecting the difficulty of differentiating diagnosis between pregnancy-specific hypertension, chronic hypertension and chronic hypertension overlapping with gestational hypertension.^26,27^ Failure to record the “HELLP syndrome” code may result from its having been more recently included in the ICD-10 with effect from 2019. 

Underreporting postpartum hemorrhage diagnosis is contrary to what is reported in the literature, whereby this diagnosis is considered to be overestimated when using ICD-10 records.^28^ A possible explanation is that the complication that gave rise to hemorrhaging was recorded in its place (e.g., uterine inertia) or that the ICD code for hemorrhaging was not recorded as hemorrhaging had been resolved. The more frequent recording of the “blood product transfusion” procedure, compared to diagnosis of “hemorrhages”, supports this hypothesis. 

It should be noted that ICD codes recorded in any of the 12 fields available for diagnosis recording were considered and not just the code in the primary diagnosis field, with the aim of achieving greater sensitivity in identifying obstetric hospitalizations and complications. In some hospital units, all diagnoses of “eclampsia” were recorded in the “ICD notification” field and not in the primary diagnosis field (data not shown), probably reflecting concern with surveillance of this condition. Using only the diagnosis recorded in the primary ICD field would result in even greater under-reporting of complications, as well as lower capture of obstetric hospitalizations.

With regard to procedures, under-reporting of major surgical interventions, such as “hysterectomy” and “laparotomy” is noteworthy, these being markers of serious complications. Regarding recording of hospitalizations in ICUs, there was an improvement in relation to a previous study, which assessed hospitalizations due to acute myocardial infarction,^29^ although the frequency found is still lower than that recorded in medical record data. Absence of accredited beds for intensive care, or even rejection of the AIH, are possible explanations for this result.

This study has limitations. Only hospitals with more than 2,750 live births per year were analyzed, making it impossible to estimate whether the same results would be found in smaller hospitals. Comparisons between the number of hospitalizations, reason for hospitalization and discharge type were made between frequencies estimated in the two databases, and it is not possible to state that the hospitalizations found relate to the same women. Finally, the MMG study, used as a reference standard, contains data obtained from hospital records that depend on record quality in each health facility. 

Notwithstanding these limitations, this is the first study dedicated to assessing obstetric hospitalizations held on the SIH/SUS and comparing them with medical record data obtained in a national study, conducted in public and private hospitals. *Episodes of care* were also analyzed, a method already adopted in previous SIH/SUS studies^30^ but not in recent studies on obstetric morbidity, thus allowing a better estimate of the number of obstetric hospitalizations and complications recorded for such hospitalizations. Finally, simultaneous analysis of the reduced database and the professional services database allowed us to identify morbidities and procedures that can be more adequately assessed using both databases.

Future studies need to investigate SIH/SUS recording in smaller hospitals and the reasons for differences in recording between different hospitals, as well as assessing strategies for improving the quality of recording on the SIH/SUS. 

The study of maternal morbidity is an important component of strategies to improve obstetric care and reduce maternal mortality. The SIH/SUS is a nationwide information system that contains morbidity data and the results of this study show a high capture of obstetric hospitalizations when using the proposed operational definitions. However, specific diagnoses, with the exception of “ectopic pregnancy”, were under-reported, as were procedures that are indicators of management of serious complications. 

Continuous improvement of AIH records, especially diagnoses and risk procedures most relevant to maternal morbidity and mortality, such as hypertensive and hemorrhagic complications, is essential for the use of the SIH/SUS in maternal morbidity surveillance. Possible strategies to be recommended include training of health professionals and implementation of standardized practices, to be followed even in exceptional periods, such as pandemics, aiming to improve accuracy and consistency in recording diagnoses and procedures, including secondary diagnoses, in both public and private hospitals. 
