# Supplementary material for: Validation study of obstetric hospitalization data held on the Brazilian National Health System Hospital Information System for maternal morbidity surveillance: Brazil, 2021-2022
Source: Epidemiol Serv Saude. 2024 Jul 29;33:e20231252. doi: 10.1590/S2237-96222024v33e20231252.en (PMC11295267; doi:10.1590/S2237-96222024v33e20231252.en)
Supplement: Translation

# Translation: Estudo de validação das internações obstétricas no Sistema de Informações Hospitalares do Sistema Único de Saúde para a vigilância da morbidade materna: Brasil, 2021-2022

**DOI:** 10.1590/S2237-96222024v33e20231252.pt

**Authors:** Rosa Maria Soares Madeira Domingues, Lana dos Santos Meijinhos, Luis Carlos Torres Guillen, Marcos Augusto Bastos Dias, Valéria Saraceni, Rejane Sobrinho Pinheiro, Natália Santana Paiva, Cláudia Medina Coeli

**Affiliations:** 1 Fundação Oswaldo Cruz, Laboratório de Pesquisa Clínica em DST/Aids, Rio de Janeiro, RJ, Brasil; 2 Universidade Federal do Rio de Janeiro, Instituto de Estudos em Saúde Coletiva, Rio de Janeiro, RJ, Brasil; 3 Fundação Oswaldo Cruz, Instituto Nacional de Saúde da Mulher, da Criança e do Adolescente “Fernandes Figueira”, Rio de Janeiro, RJ, Brasil; 4 Secretaria Municipal de Saúde do Rio de Janeiro, Superintendência de Vigilância em Saúde, Rio de Janeiro, RJ, Brasil

**Keywords:** Estudos de Validação, Morbidade, Gravidez, Período Pós-Parto, Sistemas de Informação Hospitalar, Bases de Dados Estatísticos

## Abstract

**Objetivo:**

Validar o Sistema de Informações Hospitalares do Sistema Único de Saúde (SIH/SUS) para vigilância da morbidade materna.

**Métodos:**

Estudo transversal, de 2021-2022, utilizando-se como referência dados de estudo nacional sobre morbidade materna (MMG) realizado em 50 hospitais públicos e 28 privados; foram comparados frequência, motivo e tipo de saída das internações, segundo SIH/SUS e MMG, e calculadas sensibilidade, especificidade, razão de verossimilhança positiva e negativa para sete diagnósticos e quatro procedimentos.

**Resultados:**

Internações identificadas no SIH/SUS (32.212) corresponderam a 95,1% das internações avaliadas no MMG (33.867), tendo-se observado menor registro no SIH/SUS (85,5%) em hospitais privados [10.036 (SIH/SUS); 11.742 (MMG)]; comparado ao MMG, o SIH/SUS apresentou menor proporção de internações por “intercorrências na gestação” (9,7% *versus* 16,5%), bem como sub-registro de todos os diagnósticos e procedimentos avaliados, exceto “gestação ectópica”.

**Conclusão:**

Melhor registro de diagnósticos e procedimentos no SIH/SUS é essencial para sua utilização na vigilância da morbidade materna.

## Contribuições do estudo

**Principais resultados:**

O SIH/SUS apresentou boa cobertura de registro de internações obstétricas, porém menor proporção de internações devido a “intercorrências na gestação”; e sub-registro dos diagnósticos e procedimentos avaliados, exceto “gestação ectópica”.

**Implicações para os serviços:**

É necessário melhorar a qualidade de preenchimento das autorizações de internação hospitalar (AIHs), incluindo-se o registro de diagnósticos e procedimentos para identificação da internação como obstétrica e de complicações na gravidez e puerpério.

**Perspectivas:**

A melhoria na qualidade do registro das AIHs é essencial para a utilização do SIH/SUS na vigilância da morbidade materna, estratégia recomendada para um melhor cuidado obstétrico, de forma complementar à vigilância da mortalidade materna.

## INTRODUÇÃO

A mortalidade materna é um grave problema de saúde pública, no Brasil e no mundo.^1^ Embora a razão de mortalidade materna no país seja elevada, o óbito materno é um evento pouco frequente, principalmente em locais com baixo número de nascimentos. Desde 2011, a Organização Mundial da Saúde (OMS) recomenda o estudo da morbidade materna grave e do *near miss* materno como estratégias complementares ao estudo do óbito materno, por serem eventos mais frequentes e compartilharem os mesmos fatores determinantes, permitindo análises mais robustas.^2^

O Sistema de Informações Hospitalares do Sistema Único de Saúde (SIH/SUS) tem por objetivo o pagamento de internações hospitalares com financiamento público. Se o SIH/SUS não se propõe, como uma finalidade objetiva, a vigilância em saúde, ele tem sido utilizado para investigar a morbidade hospitalar,^3^ inclusive a morbidade materna.^4-8^ Estudos anteriores,^9,10^ realizados na década de 1990, avaliaram a confiabilidade dos dados do SIH/SUS, inclusive a informação sobre parto.^11^

Todavia não foram identificados estudos que tenham avaliado nacionalmente as internações obstétricas no SIH/SUS, quando comparadas a dados de internação obtidos em prontuários hospitalares, apesar de o uso de dados administrativos para estudo da morbidade materna e neonatal ser relatado na literatura internacional.^12-14^ Analisar as informações disponíveis no SIH/SUS é, portanto, relevante para a confirmação de sua validade no estudo da morbidade materna, elaboração de estratégias de melhoria do cuidado obstétrico e redução da mortalidade materna. 

Em 2021-2022, a pesquisa nacional “Mortalidade perinatal, morbidade materna grave e *near miss* materno” (estudo MMG)^15^ avaliou a morbidade materna grave em hospitais públicos e privados brasileiros, utilizando-se de dados de prontuário coletados de forma padronizada, por profissionais treinados. 

Este estudo teve por objetivo validar a utilidade do SIH/SUS para a vigilância da morbidade materna, tendo o estudo MMG como padrão de referência, por meio da comparação da frequência, motivo e tipo de saída das internações obstétricas, e o cálculo da sensibilidade, especificidade e razão de verossimilhança positiva e negativa de diagnósticos e procedimentos recomendados pela OMS^2^ para essa vigilância. 

## MÉTODOS

### Desenho do estudo

Trata-se de estudo transversal, realizado em 2021/2022, de validação do SIH/SUS para a vigilância da morbidade materna utilizando-se como padrão de referência o estudo MMG.^15^

### Contexto

O MMG é um estudo de base hospitalar e abrangência nacional, realizado de forma integrada à pesquisa “Nascer no Brasil II: pesquisa nacional sobre aborto, parto e nascimento (NBII)”. Todas as unidades de saúde – públicas e privadas – com mais de 2.750 partos/ano e participantes do estudo NBII foram incluídas no estudo MMG. Nessas unidades, realizou-se um censo das internações obstétricas durante 30 dias consecutivos, no período 2021-2022. 

O SIH/SUS é um sistema de informações em saúde de âmbito nacional, composto por duas bases: a base reduzida, que contém os diagnósticos de internação e o procedimento médico principal realizado; e a base de serviços profissionais, com registro de todos os atos profissionais realizados durante a internação hospitalar. O número da Autorização de Internação Hospitalar (AIH) identifica a internação do mesmo indivíduo nas duas bases. 

Durante uma internação obstétrica, é possível que uma mulher tenha mais de uma AIH. Segundo as normas do SIH/SUS,^16^ deve ser emitida uma nova AIH durante a mesma internação hospitalar em algumas situações específicas, como, por exemplo, quando a mulher internada para procedimento obstétrico necessita de uma intervenção cirúrgica. Nas situações previstas,^16^ a AIH inicial deve ser encerrada com o motivo de cobrança “permanência” e uma nova AIH deve ser emitida.

### Fontes de dados

No estudo MMG, todos os dados foram provenientes de coleta de dados de prontuário, realizada de forma padronizada por profissionais treinados. O total de internações, motivo de internação, tipo de saída e diagnósticos e procedimentos médicos indicadores de morbidade materna^15^ foram obtidos na ficha de triagem, instrumento de coleta de dados preenchido para todas as mulheres incluídas no estudo. Para as mulheres com morbidade registrada na ficha de triagem, realizou-se coleta de dados adicional, utilizando-se um instrumento detalhado. 

No SIH/SUS, o total de internações, motivo de internação, tipo de saída e diagnósticos de internação foram obtidos na base reduzida, enquanto procedimentos realizados foram obtidos na base reduzida e na base de serviços profissionais. Tanto a base reduzida como a base de serviços profissionais foram acessadas em 15 de agosto de 2022. 

### Participantes

No estudo MMG, foram incluídos 78 hospitais – 50 públicos e 28 privados, que haviam concluído a coleta de dados até fevereiro de 2022 –, distribuídos em todas as Unidades da Federação, exceto o Amapá. 

No SIH/SUS, foram incluídas todas as internações obstétricas ocorridas no mesmo período do estudo MMG, em cada um dos hospitais participantes, estes identificados por seu número do Cadastro Nacional de Estabelecimentos de Saúde (CNES). O processo de seleção das internações teve quatro etapas (Figura 1). Inicialmente, foram selecionadas AIHs do tipo 1 de mulheres de 10 a 49 anos de idade, na base reduzida, não identificada, disponível no *site* do Departamento de Informática do SUS (Datasus) e captada pelo pacote Microdatasus,^17^ utilizando-se a linguagem de programação estatística R. 

**Figura 1:** Procedimentos adotados para seleção das internações obstétricas no Sistema de Informações Hospitalares do Sistema Único de Saúde, Brasil, 2021-2022

Na segunda etapa do processo seletivo, foi identificado o *episódio de cuidado* hospitalar^18^ de cada mulher, definido como o conjunto de todas as informações referentes a determinada internação hospitalar, podendo ser constituído por uma ou múltiplas AIHs. Para identificação de um *episódio de cuidado* formado por múltiplos registros de AIHs, utilizou-se um algoritmo que permitiu a identificação de AIHs subsequentes a uma AIH com motivo de cobrança “permanência”. De forma resumida, AIHs com o mesmo número do CNES e cuja mulher apresentava a mesma data de nascimento foram consideradas como sendo parte de um mesmo *episódio de cuidado*, se o intervalo entre a data de saída de uma AIH com motivo de cobrança “permanência” e a data de internação da AIH subsequente fosse menor ou igual a um dia. 

Posteriormente, para a identificação das internações obstétricas, foram selecionadas as AIHs com diagnósticos de internação [segundo a Classificação Estatística Internacional de Doenças e Problemas Relacionados à Saúde – Décima Revisão (CID-10)] e/ou procedimentos do Sistema de Gerenciamento da Tabela de Procedimentos, Medicamentos e OPM (OPM: órteses, próteses e materiais especiais) do SUS^19^ descritos no Quadro 1, anexo a este relato. Nesta seleção, foram considerados os *episódios de cuidado* e identificadas AIHs com critério para internação obstétrica em *episódios de cuidado* com registro único ou múltiplos registros, independentemente da posição da AIH no episódio. Finalmente, após a identificação das internações obstétricas na base reduzida, AIHs com mesmo número foram selecionadas na base de serviços profissionais.

**Quadro 1:** Definições operacionais utilizadas na base do Sistema de Informações Hospitalares do Sistema Único de Saúde para classificação de internação obstétrica, motivo de internação, tipo de saída, diagnósticos e procedimentos específicos

**Critério**	**CID** ^a^		**Procedimentos** ^b^
**Internação obstétrica**	CID do grupo “O” registrada em um dos seguintes campos: diagnóstico principal, diagnóstico secundário, diagnósticos secundários 1 a 9, CID associada, CID notificação, CID óbito	OU	02.01.01.001-1, 02.11.04.001-0, 02.11.04.006-1, 03.10.01.001-2, 03.10.01.002-0, 03.10.01.003-9, 03.10.01.004-7, 03.10.01.005-5, 03.03.10.001-0, 03.03.10.002-8, 03.03.10.003-6, 03.03.10.004-4, 03.03.10.005-2, 04.09.06.001-1, 04.09.06.005-4, 04.09.06.007-0, 04.11.01.001-8, 04.11.01.002-6, 04.11.01.003-4, 04.11.01.004-2, 04.11.01.005-0, 04.11.01.007-7, 04.11.01.008-5, 04.11.02.001-3, 04.11.02.002-1, 04.11.02.003-0, 04.11.02.004-8, 04.17.01.002-8, 04.17.01.001-0, 04.17.01.003-6
**Motivo de internação**			
Aborto	CID principal O00 a O08		
Parto	CID principal 032 a 036, O60 a O69, O75, O80 a O84, P95	OU	03.10.01.001-2, 03.10.01.003-9, 03.10.01.004-7, 03.10.01.005-5, 04.11.01.002-6, 04.11.01.003-4, 04.11.01.004-2
Intercorrências na gestação	CID principal O10 a O28, O30, O31, O40-O48 ou qualquer CID de outro grupo, desde que o procedimento solicitado seja diferente de 03.10.01.001-2, 03.10.01.003-9, 03.10.01.004-7, 03.10.01.005-5, 04.11.01.002-6, 04.11.01.003-4, 04.11.01.004-2	OU	03.03.10.004-4
Intercorrências no puerpério	CID principal O70, O71, O72, O73, O85 a O94		03.03.10.001-0
**Tipo de saída**			
Alta	Motivo cobrança = 1, 6.1, 6.2, 6.3, 6.4		
Administrativa	Motivo cobrança = 5		
Permanência	Motivo cobrança = 2		
Morte	Motivo cobrança = 4, 6.5, 6.6, 6.7		
Transferência	Motivo cobrança = 3		
Em branco	Motivo cobrança não preenchido		
**Diagnósticos e procedimentos específicos**			
Pré-eclâmpsia grave	O141		
Eclâmpsia	O150, O151, O152, O159	OU	03.03.10.002-8
Síndrome HELLP^c^	O142		
Descolamento prematuro de placenta (DPP)	O450, O458, O459		
Hemorragia pós-parto/pós aborto	O720, O721, O722, O723, O031, O036, O041, O046, O051, O056, O061, O066, O071, O076, O081		
Rotura uterina	O710 e O711		
Gestação ectópica	O000, O001, O002, O008, O009	OU	04.11.02.004-8
Histerectomia	O822	OU	04.11.02.003-0, 04.09.06.010-0, 04.09.06.011-9, 04.09.06.012-7, 04.09.06.013-5
Laparotomia			04.07.04.016-1
Transfusão de hemoderivados	Z513	OU	03.06.02.006-8, 03.06.02.007-6, 03.06.02.008-4, 03.06.02.009-2, 03.06.02.010-6, 03.06.02.011-4, 03.06.02.012-2, 03.06.02.013-0, 03.06.02.014-9
Internação em UTI^d^			Variável UTI_MES_TO^e^ > 0 ou procedimentos 08.02.01.010-5, 08.02.01.008-3, 08.02.01.009-1, 08.02.01.029-6, 08.02.01.031-8

a) CID: Classificação Estatística Internacional de Doenças e Problemas Relacionados à Saúde; b) Código do procedimento realizado, segundo tabela SIGTAP (Sistema de Gerenciamento da Tabela de Procedimentos, Medicamentos e OPM do SUS); c) Síndrome HELLP: forma grave de pré-eclâmpsia, caracterizada por hemólise, enzimas hepáticas elevadas e plaquetopenia, em pacientes gestantes ou puérperas; d) UTI: Unidade de tratamento intensivo; e) UTI_MES_TO: Número de dias de internação em UTI.

### Variáveis 

As variáveis de interesse do estudo foram:

a) Tipo de hospital (público; privado)
b) Número total de internações obstétricas
c) Motivo de internação (parto; aborto; intercorrências na gestação; intercorrências no puerpério)
d) Tipo de saída (alta; administrativa; permanência; morte; transferência)
e) Diagnósticos obstétricos
  – Pré-eclâmpsia grave
  – Eclâmpsia
  – Síndrome HELLP (hemólise, enzimas hepáticas elevadas, baixa contagem de plaquetas – *Hemolysis, Elevated Liver enzymes, Low Platelet count*, HELLP)
  – Descolamento prematuro de placenta (DPP)
  – Hemorragia pós-parto/pós-aborto
  – Rotura uterina
  – Gestação ectópica
f) Procedimentos hospitalares
  – Histerectomia
  – Laparotomia
  – Transfusão de hemoderivados
  – Internação em unidade de tratamento intensivo (UTI)

Os sete diagnósticos e quatro procedimentos listados nos itens “e” e “f” fazem parte dos 26 critérios recomendados pela OMS para estudo da morbidade materna grave.^2^

### Detalhamento das variáveis

No estudo MMG, foram classificadas como internações por “intercorrências na gestação” todas aquelas devidas a complicações clínicas e/ou obstétricas ocorridas durante a gestação, sem indicação de interrupção da gestação no momento da admissão hospitalar; e como “intercorrências no puerpério”, todas as internações decorrentes de complicações clínicas e/ou obstétricas diagnosticadas após o término da gestação. Para o tipo de saída, considerou-se como “permanência” uma mulher que permaneceu internada após o 42º dia do término da gestação. Para diagnósticos e procedimentos, foram considerados os registros feitos no prontuário, sem classificação pela equipe de pesquisa. A ausência de registro foi considerada como ausência do diagnóstico e não realização do procedimento. 

No SIH/SUS, para classificação do motivo de internação e do tipo de saída, foram utilizados os critérios descritos no Quadro 1. Foi classificado como tipo de saída “permanência” um *episódio de cuidado* cuja última AIH teve alta por permanência, não sendo possível identificar a AIH subsequente nesse *episódio de cuidado*. No caso de *episódios de cuidado* com mais de uma AIH, foi utilizado o “motivo de internação” registrado na primeira AIH do *episódio de cuidado* e o “tipo de saída” registrado na última AIH do *episódio de cuidado*. Para a identificação dos diagnósticos e procedimentos, foram utilizados os códigos da CID e os procedimentos descritos no Quadro 1, sendo considerados os registros de todas as AIHs do *episódio de cuidado*. No caso de diagnóstico ou procedimento específico registrado em mais de uma AIH do *episódio de cuidado*, o diagnóstico e/ou procedimento foi contabilizado uma única vez. 

### Métodos estatísticos

Todas as análises foram realizadas comparando-se dados das internações no SIH/SUS (fonte a ser validada) com os dados de internações apresentados no estudo MMG (padrão de referência), para o conjunto dos hospitais e por hospitais públicos e hospitais privados, utilizando-se a linguagem de programação estatística R versão 4.3.0.^20^

Inicialmente, foram comparados a frequência de internações obstétricas, os motivos de internação e os tipos de saída identificados pelo estudo MMG e no SIH/SUS. Na comparação do total de internações, considerou-se adequada uma cobertura de registro igual ou superior a 90%, percentual utilizado pelo Ministério da Saúde brasileiro como parâmetro para a cobertura de registro em outros sistemas de informações.^21^ A variável “motivo de internação” foi incluída na ficha de triagem do estudo MMG após o início do trabalho de campo, sendo excluídos desta análise os hospitais com mais de 10% das internações sem essa variável.

Posteriormente, foram comparados os diagnósticos e procedimentos específicos nas duas bases. Para esta análise, foram incluídas apenas as mulheres do estudo MMG que apresentaram alguma morbidade. Foi feita a vinculação determinística dessas internações com o SIH/SUS, sendo utilizadas as informações disponíveis nas duas bases [número do CNES; data de nascimento; data de internação; data de alta; raça/cor da pele; código de endereçamento postal (CEP) da residência]. Nas internações obstétricas vinculadas, foi feita a comparação da frequência de diagnósticos e procedimentos específicos, bem como o cálculo da sensibilidade, da especificidade, e da razão de verossimilhança positiva (RV positiva) e negativa (RV negativa) de cada diagnóstico e procedimento específico no SIH/SUS. A RV positiva (sensibilidade/1 - especificidade) refere-se à presença de diagnóstico; e a RV negativa (1 - sensibilidade/especificidade), à ausência de diagnóstico. Valores superiores a 1 aumentam a probabilidade de diagnóstico e valores entre 0 e 1 reduzem essa probabilidade. Para interpretação, foram utilizados os seguintes critérios:

a) RV positiva: > 10 = forte; de 5 a 9,9 = moderada; de 2 a 4,9 = fraca; de 1 a 2 = muito fraca.
b) RV negativa: < 0,1 = forte; de 0,11 a 0,20 = moderada; de 0,21 a 0,50 = fraca; de 0,51 a 1 = muito fraca.^22^

Todos os códigos utilizados estão disponíveis no seguinte endereço eletrônico: http://github.com/coelicm/SIH-SUS-validation

### Considerações éticas

O projeto do estudo MMG foi aprovado pelo Comitê de Ética em Pesquisa da Escola Nacional de Saúde Pública Sergio Arouca/Fundação Oswaldo Cruz (CEP/ENSP/Fiocruz), sob o Parecer n^o^ 4.230.028, emitido em 21 de agosto de 2020, com emenda aprovada mediante o Parecer nº 4.473.968, emitido em 18 de dezembro de 2020. Por se tratar de estudo retrospectivo com coleta de dados de prontuário, solicitou-se a dispensa da assinatura do Termo de Consentimento Livre e Esclarecido, sendo o acesso aos prontuários autorizado pela unidade hospitalar. Todos os cuidados foram adotados visando garantir o sigilo e a confidencialidade das informações. Para a análise com dados do SIH/SUS, foram utilizadas bases não identificadas, de acesso público. 

### RESULTADOS

No estudo MMG, foram registradas 33.867 internações obstétricas, das quais 5.379 apresentavam o registro de alguma morbidade materna, sendo coletados dados detalhados de 5.303 prontuários. No SIH/SUS, foram identificadas 32.212 internações obstétricas nos mesmos hospitais e período do estudo, sendo 4.652 relacionadas às internações do estudo MMG que apresentavam diagnóstico de morbidade (Figura 2). 

**Figura 2:** Fluxograma de internações segundo o estudo Morbidade Materna Grave e o Sistema de Informações Hospitalares do Sistema Único de Saúde, Brasil, 2021-2022

O número de internações obstétricas captadas no SIH/SUS correspondeu a 95,1% do número de internações avaliadas no estudo MMG, sendo essa proporção de 100,2% em hospitais públicos (22.176/22.125) e de 85,5% em hospitais privados (10.036/11.742). Entretanto, a frequência de registro variou entre os hospitais: quando os dados do SIH/SUS foram comparados aos do estudo MMG, 8% dos hospitais públicos e 50% dos hospitais privados apresentaram proporção de registro inferior a 90%, enquanto 14% dos hospitais públicos e 10,7% dos hospitais privados apresentaram proporção de registro de internação superior a 110%. 

Verificou-se uma proporção maior de internações por “intercorrências na gestação” no estudo MMG, tanto nos hospitais públicos (16,1% *versus* 9,9%) como nos hospitais privados (17,4% *versus* 9,1%), e uma proporção maior de internações para parto no SIH/SUS (78,7% *versus* 72,2% em hospitais públicos; 81,2% *versus* 73,0% em hospitais privados). Os motivos de saída no estudo MMG e no SIH/SUS foram semelhantes (Tabela 1), destacando-se que “permanência” teve interpretações diferentes nas duas bases. 

**Tabela 1:** Motivo de internação obstétrica e tipo de alta hospitalar segundo o estudo Morbidade Materna Grave e o Sistema de Informações Hospitalares do Sistema Único de Saúde, Brasil, 2021-2022

**Motivo de internação**	**Unidades públicas**		**Unidades privadas**		**Total**	
	MMG ^a^ (n = 18.688) ^c^ (%)	SIH/SUS ^b^ (n = 18.911) (%)	MMG ^a^ (n = 8.615) ^c^ (%)	SIH/SUS ^b^ (n = 7.177) (%)	MMG ^a^ (n = 27.303) ^c^ (%)	SIH/SUS ^b^ (n 26.088) (%)
Parto	72,2	78,7	73,0	81,2	72,5	79,4
Aborto	9,2	8,7	7,7	7,3	8,8	8,3
Intercorrência na gestação	16,1	9,9	17,4	9,1	16,5	9,7
Intercorrência no puerpério	2,3	2,5	1,8	1,7	2,1	2,3
Outros^d^	–	0,2	–	0,7	–	0,4
Em branco^e^	0,1	–	0,1	–	0,1	–
**Tipo de saída**	**Unidades públicas**		**Unidades privadas**		**Total**	
	**MMG** ^a^ **(n = 22.125)** **(%)**	**SIH/SUS** ^b^ **(n = 22.176)** **(%)**	**MMG** ^a^ **(n = 11.742)** **(%)**	**SIH/SUS** ^b^ **(n = 10.036)** **(%)**	**MMG** ^a^ **(n =33.867)** **(%)**	**SIH/SUS** ^b^ **(n = 32.212)** **(%)**
Alta	96,7	97,0	98,1	99,0	97,2	97,6
Administrativa	1,4	0,6	1,0	–	1,3	0,5
Permanência^f^	–	0,9	–	0,3	–	0,7
Morte	–	0,1	0,1	0,1	0,1	0,1
Transferência	1,9	1,4	0,7	0,5	1,5	1,1

a) MMG: Estudo “Mortalidade perinatal, morbidade materna grave e *near miss* materno”; b) SIH/SUS: Sistema de Informações Hospitalares do Sistema Único de Saúde; c) Excluídas as unidades hospitalares com proporção de internação “não preenchido” > 10% no estudo MMG; d) “Outros” refere-se a outros diagnósticos de internação não classificados como parto, aborto, intercorrências na gestação ou intercorrência no puerpério; e) “Em branco” corresponde a casos sem informação do motivo de internação; f) No estudo MMG, o tipo de saída “permanência” refere-se a mulheres que permaneciam internadas após o 42º dia pós-parto ou pós-aborto, enquanto no SIH/SUS o tipo de saída “permanência” refere-se ao motivo de cobrança “permanência”, utilizado quando a mulher apresenta algum critério que exige emissão de nova autorização de internação hospitalar (AIH) na mesma internação hospitalar, não tendo sido identificada a AIH subsequente.

A análise dos diagnósticos e procedimentos específicos (Tabela 2) mostrou que os diagnósticos mais frequentes no estudo MMG foram “pré-eclâmpsia grave” (17,7%) e “hemorragia” (13,8%), sendo “internação em UTI” o procedimento mais frequente (7,1%). No SIH/SUS, os diagnósticos mais frequentes foram “pré-eclâmpsia grave” (8,9%) e “gestação ectópica” (4,2%); e entre os procedimentos, os mais frequentes foram “internação em UTI” (5,3%) e “transfusão de hemoderivados” (5,2%). Destaca-se a ausência de casos de síndrome HELLP no SIH/SUS e a identificação dos procedimentos relativos a “transfusão de hemoderivados” e “internação em UTI” exclusivamente na base de serviços profissionais, sendo possível a identificação de “internação em UTI” na base reduzida por meio da variável “UTI_MES_TO”.

Dois diagnósticos (“eclâmpsia” e “DPP”) e três procedimentos (“laparotomia”, “transfusão de hemoderivados” e “internação em UTI”) apresentaram RV positiva “forte” em hospitais públicos e privados, enquanto “pré-eclâmpsia grave”, “rotura uterina” e “gestação ectópica” também apresentaram RV positiva “forte” em hospitais públicos. “Hemorragia pós-parto ou pós-aborto” apresentou RV positiva “moderada” em hospitais públicos e “muito fraca” em hospitais privados. Todos os diagnósticos e procedimentos apresentaram RV negativa “muito fraca” em hospitais públicos e privados, exceto “gestação ectópica” (“forte” em hospitais públicos e privados), “transfusão de hemoderivados” (“fraca” em hospitais privados) e “internação em UTI” (“fraca” em hospitais públicos e “moderada” em hospitais privados) (Tabela 2). 

**Tabela 2:** Frequência, sensibilidade, especificidade e razão de verossimilhança negativa e positiva de diagnósticos específicos e procedimentos em internações obstétricas, por caráter do hospital, segundo o Sistema de Informações Hospitalares do Sistema Único de Saúde, Brasil, 2021-2022

**Diagnósticos e procedimentos específicos**	**Fonte dos dados**					**Sens.** ^g^ **(%)**	**Espec.** ^h^ **(%)**	**RV neg** ^i^	**RV pos** ^j^
	**MMG** ^a^ **n (%)**	**SIH/SUS** ^b^							
		**CID** ^c^ **n (%)**	**PROC_REA** ^d^ **n (%)**	**ATO PROF** ^e^ **n (%)**	**TOTAL** ^f^ **n (%)**				
**Diagnósticos**									
**Pré-eclâmpsia grave**									
Hospital público	639 (18,3)	317 (9,1)	–	–	317 (9,1)	39,3	97,7	0,62	17,0
Hospital privado	184 (15,9)	97 (8,4)	–	–	97 (8,4)	33,7	96,4	0,69	9,4
Total	823 (17,7)	414 (8,9)			414 (8,9)	38,0	97,4	0,64	14,4
**Eclâmpsia**									
Hospital público	94 (2,7)	71 (2,0)	4 (0,1)	4 (0,1)	71 (2,0)	20,2	98,5	0,81	13,2
Hospital privado	32 (2,8)	15 (1,3)	–	–	15 (1,3)	31,3	99,6	0,69	70,3
Total	126 (2,7)	86 (1,8)	4 (0,1)	4 (0,1)	86 (1,8)	23,0	98,7	0,78	18,3
**Sínd. HELLP** ^k^									
Hospital público	105 (3,0)	–	–	–	–		100,0	1,0	–
Hospital privado	40 (3,5)	–	–	–	–		100,0	1,0	–
Total	145 (3,1)	–			–	–	100,0	1,0	–
**DPP** ^l^									
Hospital público	129 (3,7)	59 (1,7)	–	–	59 (1,7)	42,6	99,9	0,57	358,9
Hospital privado	47 (4,1)	16 (1,4)	–	–	16 (1,4)	25,5	99,6	0,75	70,8
Total	176 (3,8)	75 (1,6)			75 (1,6)	38,1	99,8	0,62	213,0
**Hemorragias**									
Hospital público	466 (13,3)	53 (1,5)	–	–	53 (1,5)	5,2	99,0	0,96	5,4
Hospital privado	175 (15,1)	22 (1,9)	–	–	22 (1,9)	2,9	98,3	0,99	1,7
Total	641 (13,8)	75 (1,6)			75 (1,6)	4,5	98,9	0,97	3,9
**Rotura uterina**									
Hospital público	23 (0,7)	2 (0,1)	–	–	2 (0,1)	4,3	100,0	0,96	151,0
Hospital privado	1 (0,1)	1 (0,1)	–	–	1 (0,1)	–	99,9	1,0	–
Total	24 (0,5)	3 (0,1)			3 (0,1)	4,2	100,0	0,96	96,4
**Gestação ectópica**									
Hospital público	169 (4,8)	157 (4,5)	121 (3,5)	121 (3,5)	159 (4,5)	93,5	100,0	0,07	3.110,5
Hospital privado	41 (3,5)	33 (2,9)	30 (2,6)	31 (2,7)	37 (3,2)	90,2	100,0	0,10	–
Total	210 (4,5)	190 (4,1)	151 (3,2)	152 (3,3)	196 (4,2)	92,2	100,0	0,07	4.124,7
**Procedimentos específicos**									
**Histerectomia**									
Hospital público	40 (1,1)	1 (0,0)	9 (0,3)	8 (0,2)	15 (0,4)	37,5	100,0	0,62	–
Hospital privado	23 (2,0)	–	4 (0,3)	6 (0,5)	8 (0,7)	34,8	100,0	0,65	–
Total	63 (1,4)	1 (0,0)	13 (0,3)	14 (0,3)	23 (0,5)	36,5	100,0	0,63	–
**Laparotomia**									
Hospital público	168 (4,8)	–	28 (0,8)	28 (0,8)	28 (0,8)	16,1	100,0	0,84	534,9
Hospital privado	44 (3,8)	–	6 (0,5)	6 (0,5)	6 (0,5)	11,4	99,9	0,89	126,4
Total	212 (4,6)		34 (0,7)	34 (0,7)	34 (0,7)	15,1	100,0	0,85	335,1
**Transfusão de hemoderivados**									
Hospital público	187 (5,3)	–	–	162 (4,6)	162 (4,6)	46,0	97,7	0,55	20,0
Hospital privado	73 (6,3)	–	–	80 (6,9)	80 (6,9)	61,6	96,8		19,1
Total	260 (5,6)	–	–	242 (5,2)	242 (5,2)	50,4	97,5	0,51	22,4
**Internação em UTI** ^m^									
Hospital público	243 (7,0)	168 (4,8)^n^	–	158 (4,5)	168 (4,8)	65,0	99,7	0,35	211,5
Hospital privado	86 (7,4)	80 (6,9)^n^	–	71 (6,1)	80 (6,9)	86,0	99,4	0,14	153,5
Total	329 (7,1)	248 (5,3)^n^	–	229 (4,9)	248 (5,3)	70,5	99,6	0,30	190,5

a) MMG: Estudo “Mortalidade perinatal, morbidade materna grave e *near miss* materno”; b) SIH/SUS: Sistema de Informações Hospitalares do Sistema Único de Saúde; c) CID: Classificação Estatística Internacional de Doenças e Problemas Relacionados à Saúde; d) PROC_REA: Procedimento realizado registrado na base reduzida do SIH/SUS; e) ATO PROF: Procedimento realizado registrado na base de serviços profissionais do SIH/SUS; f) TOTAL: Diagnóstico considerando registro em CID, PROC-REA e ATO PROF; g) Sens.: Sensibilidade; h) Espec.: Especificidade; i) RV neg.: Razão de verossimilhança negativa; j) RV pos.: Razão de verossimilhança positiva; k) Sínd.HELLP: Síndrome HELLP (forma grave de pré-eclâmpsia caracterizada por hemólise, enzimas hepáticas elevadas e plaquetopenia em pacientes gestantes ou puérperas); l) DPP: Descolamento prematuro de placenta; m) UTI: Unidade de tratamento intensivo; n) Internação em UTI, identificada na base reduzida por meio da variável “UTI_MES_TO”, que contém a informação sobre o número de dias de internação em UTI. Total de internações em unidade públicas: 3.496, e total de internações em unidades privadas: 1.156.

## DISCUSSÃO

Os resultados encontrados mostraram elevada captação de internações obstétricas no SIH/SUS, principalmente em hospitais públicos, com proporções semelhantes quanto ao tipo de saída e motivo de internação, na comparação entre os registros do SIH/SUS e os registros do estudo MMG; à exceção das internações por “intercorrências na gestação”, que apresentaram proporção 40% menor no SIH/SUS. Oito diagnósticos e procedimentos apresentaram RV positiva “forte” em hospitais públicos e cinco em hospitais privados, demonstrando a elevada probabilidade desses diagnósticos e procedimentos quando registrados no Sistema. Entretanto, todos os diagnósticos e procedimentos, à exceção de “gestação ectópica”, apresentaram evidência de sub-registro, limitando o uso do SIH/SUS para a vigilância da morbidade materna. 

O menor número de internações observado em hospitais privados seria um resultado esperado, já que nem todos os leitos de um hospital privado conveniado ao SUS realizam internações com financiamento público. A variação na proporção de registro observada nos hospitais avaliados suscita algumas possíveis explicações, tais como (i) a não emissão de AIH, (ii) a não autorização (de AIH) emitida, ou ainda, (iii) a emissão de AIH sem diagnósticos do grupo “O” ou procedimentos utilizados para a identificação de internações obstétricas. 

Em internações que apresentam diagnóstico principal e procedimentos por causas não obstétricas, como pode ter ocorrido em internações por COVID-19, é fundamental que códigos/CID do grupo “O” relacionados a intercorrências na gestação ou no puerpério sejam registrados nos diagnósticos secundários, para que se possa identificar essas internações como tendo-se realizado durante a gestação ou puerpério. 

Quanto ao maior número de AIHs identificadas no SIH/SUS, as hipóteses explicativas incluem (i) a não identificação de uma AIH como parte de um *episódio de cuidado*, sendo contabilizada como uma internação de outra mulher, (ii) a não captação de internação de gestantes e puérperas pelo estudo MMG, caso mulheres com diagnóstico de COVID-19 tenham-se internado fora da maternidade (por exemplo, em leitos de emergência ou de isolamento respiratório), e (iii) a emissão de AIH para internações não realizadas.

O resultado de menor proporção de internações por “intercorrências na gestação” no SIH/SUS pode-se atribuir à forma de classificação do motivo de internação nas duas bases: no SIH/SUS, mulheres com diagnóstico de uma complicação na gravidez na internação foram classificadas como “parto” se o procedimento realizado foi um dos previstos para assistência ao parto normal ou realização de cesariana. Cumpre observar as proporções muito semelhantes de internações para assistência ao aborto, indicando não haver sub-registro de internações por abortamento, o que seria possível, considerando-se a ilegalidade e estigmatização do tema no país.

A maioria das saídas foi por alta hospitalar, sendo a proporção de óbitos baixa e semelhante entre as duas bases. Um estudo de avaliação da mortalidade materna no Brasil em 2019, estimada por meio de dados registrado no SIH/SUS, concluiu que o uso do SIH/SUS pode ser válido em estudos sobre mortalidade e morbidade materna, enquanto um sistema de informações complementar ao Sistema de Informação sobre Mortalidade (SIM).^23^

O sub-registro de diagnósticos e procedimentos na base SIH/SUS tem implicações para o estudo da morbidade materna, principalmente das complicações hipertensivas e hemorrágicas, causas mais frequentes de morbidade materna encontradas tanto no estudo MMG como em estudos nacionais^4,6,8^ e internacionais.^24,25^ Problemas no registro de diagnóstico específicos no SIH/SUS já haviam sido relatados em estudos anteriores.^3^

Para o diagnóstico “pré-eclâmpsia grave”, é possível que se tenham utilizado outros códigos da CID relacionados a complicações hipertensivas, refletindo a dificuldade do diagnóstico diferencial entre a hipertensão específica da gestação, hipertensão crônica e hipertensão crônica superposta com hipertensão gestacional.^26,27^ Já a ausência de registro do código da “síndrome HELLP” pode resultar de sua inclusão mais recente na CID-10, adotada em 2019. 

O sub-registro do diagnóstico de hemorragia pós-parto é contrário ao relatado na literatura, onde considera-se que esse diagnóstico esteja superestimado ao usar registros da CID-10.^28^ Uma possível explicação é que tenha sido registrada a complicação que deu origem à hemorragia (por exemplo, atonia uterina) ou que não tenha havido registro da CID de hemorragia uma vez que a mesma tenha sido resolvida. O registro mais frequente do procedimento “transfusão de hemoderivados”, comparado ao diagnóstico “hemorragia”, reforça essa hipótese. 

Ressalta-se que foram considerados códigos da CID registrados em qualquer um dos 12 campos disponíveis para registro de diagnóstico e não apenas no campo de diagnóstico principal, visando maior sensibilidade na identificação das internações e complicações obstétricas. Em algumas unidades hospitalares, todos os diagnósticos de “eclâmpsia” estavam registrados no campo “CID notificação” e não no diagnóstico principal (dado não mostrado em tabela), provavelmente reflexo de uma preocupação com a vigilância desse agravo. A utilização apenas do diagnóstico registrado na CID principal resultaria em sub-registro ainda maior de complicações, como também menor captação de internações obstétricas.

Para os procedimentos, cabe destacar o sub-registro de intervenções cirúrgicas de grande porte, como “histerectomia” e “laparotomia”, marcadoras de complicações graves. No que concerne ao registro de internações em UTIs, houve melhora em relação a estudo anterior, que avaliou internações por infarto agudo do miocárdio,^29^ embora a frequência observada ainda seja inferior à registrada em dados de prontuário. A ausência de leitos credenciados para tratamento intensivo, ou mesmo a rejeição da AIH, são explicações possíveis para esse resultado.

O presente estudo tem algumas limitações. Foram analisados apenas hospitais com mais de 2.750 nascidos vivos por ano, não sendo possível estimar se os mesmos resultados seriam observados em hospitais de menor porte. As comparações entre número de internações, motivo de internação e tipo de saída foram feitas entre frequências estimadas nas duas bases, não sendo possível afirmar que as internações observadas sejam das mesmas mulheres. Finalmente, o estudo MMG, utilizado como padrão de referência, contém dados obtidos do prontuário hospitalar que dependem da qualidade do registro em cada serviço. 

Não obstante essas limitações, trata-se aqui do primeiro estudo dedicado à avaliação de internações obstétricas no SIH/SUS e sua comparação com dados de prontuário obtidos em estudo nacional, realizado em hospitais públicos e privados, com coleta de dados padronizada por profissionais treinados. Também foi utilizada a análise dos *episódios de cuidado* (e não de AIH individualmente), já adotada em estudos anteriores com o SIH/SUS^30^ mas não em estudos recentes sobre morbidade obstétrica, permitindo melhor estimativa do número de internações obstétricas e das complicações registradas nessas internações. Por fim, a análise simultânea da base reduzida e da base de serviços profissionais permitiu identificar as morbidades e procedimentos que podem ser avaliados de forma mais adequada com o uso das duas bases.

Estudos futuros devem investigar o registro do SIH/SUS em hospitais de menor porte e as razões para diferenças de registro entre diferentes hospitais, bem como avaliar estratégias de melhoria da qualidade do registro no Sistema. 

O estudo da morbidade materna é um importante componente das estratégias de melhoria da atenção obstétrica e de redução da mortalidade materna. O SIH/SUS é um sistema de informação de abrangência nacional que contém dados de morbidade e os resultados deste estudo mostram elevada captação de internações obstétricas ao utilizar as definições operacionais propostas. Entretanto, diagnósticos específicos, à exceção de “gestação ectópica”, apresentaram sub-registro, tal qual procedimentos indicadores de manejo de complicações graves. 

A melhoria contínua dos registros nas AIHs, especialmente dos diagnósticos e procedimentos de risco mais relevantes para a morbimortalidade materna, como complicações hipertensivas e hemorrágicas, é essencial para a utilização do SIH/SUS na vigilância da morbidade materna. Possíveis estratégias a recomendar incluem o treinamento de profissionais e a implementação de práticas padronizadas, para serem seguidas mesmo em períodos excepcionais, como pandemias, visando melhorar a precisão e a consistência no registro de diagnósticos e procedimentos, incluindo diagnósticos secundários, tanto em hospitais públicos como em hospitais privados.
